# Mechanisms and clinical implications of gut-brain interactions

**DOI:** 10.1172/JCI196346

**Published:** 2026-01-02

**Authors:** Zachary S. Lorsch, Rodger A. Liddle

**Affiliations:** 1 Department of Medicine and; 2 Duke Institute for Brain Sciences, Duke University, Durham, North Carolina, USA.; 3 Department of Veterans Affairs Healthcare System, Durham, North Carolina, USA.

## Abstract

Connections between the digestive system and the brain have been postulated for over 2000 years. Despite this, only recently have specific mechanisms of gut-brain interaction been identified. Due in large part to increased interest in the microbiome, the wide use of incretin-based therapies (i.e., glucagon-like peptide 1 [GLP-1] receptor agonists), technological advancements, increased understanding of neuroimmunology, and the identification of a direct enteroendocrine cell–neural circuit, research in the past 10 years has made it abundantly clear that the gut-brain connection plays a role both in clinical disease as well as the actions of therapeutics. In this Review, we describe mechanisms by which the gut and brain communicate and highlight human and animal studies that implicate changes in gut-brain communication in disease states in gastroenterology, neurology, psychiatry, and endocrinology. Furthermore, we define how GLP-1 receptor agonists for obesity and guanylyl cyclase C agonists for irritable bowel syndrome leverage gut-brain mechanisms to improve patient outcomes. This Review illustrates the critical nature of gut-brain communication in human disease and the potential to target gut-brain pathways for therapeutic benefit.

## Introduction

A common phrase attributed to the ancient Greek physician Hippocrates is, “all diseases begin in the gut.” While this gut-centric approach to medicine did not persist, recent research has implicated interactions between the digestive system, the gut microbiome, and other organ systems in wide-ranging pathophysiologies, including heart (1–3), lung (4), and chronic kidney diseases (5–7), cerebrovascular abnormalities (8), and cancer (9, 10). Due in part to a defined signaling pathway between the two organs via the vagus nerve (11), research on communication between the gastrointestinal tract and brain has given rise to the new field of gut-brain interactions.

Recognition of bidirectional communication between the gut and the brain is not new, as numerous historical examples, including William Beaumont’s 1833 finding that emotional arousal affects gastric function (12) and Ivan Pavlov’s Nobel Prize–winning work on the cephalic phase of digestion (13), suggested a physiologic connection between the two organs. However, only recently have the pathways and mechanisms underlying gut-brain interactions been described, and an array of disease phenotypes in which altered gut-brain communication is directly implicated been discovered. Furthermore, with the increasing use of incretin-based therapies (i.e., glucagon-like peptide 1 [GLP-1] receptor agonists) that leverage gut-brain mechanisms, gut-brain communication has become an important topic in both clinical and translational medicine. Here, we describe the four main mechanisms of communication between the gut and brain: hormonal, microbiome-mediated, immune-mediated, and direct signaling. We evaluate the role of disordered gut-brain interactions in the pathophysiology of several diseases (Table 1) and explain how the GLP-1 receptor agonists (e.g., semaglutide) for obesity and guanylyl cyclase C agonists (e.g., linaclotide) for irritable bowel syndrome (IBS) work through a gut-brain mechanism.

## Mechanisms of gut-brain interaction

Interactions between the gut and the brain are bidirectional, involving multiple communicating systems including the gut and its contents, the microbiome, peripheral nerves, the enteric nervous system (ENS), autonomic nervous system, and the brain (14). Innervation of the gut is complex, with transcriptional profiles suggesting 21 neuronal and 3 glial cell subtypes in the ENS alone (15). The basic structure of the ENS consists of a submucosal plexus that controls mucosal processes and a myenteric plexus that controls movement, both of which are supported by interneurons and glia that refine function (16). In addition, intrinsic primary afferent neurons that project from the myenteric plexus to the subepithelial space receive sensory input from the gut epithelium via neurotransmitters (17). The colon is capable of peristalsis ex vivo, illustrating that extrinsic innervation is not required for the ENS to function. However, in vivo, the ENS is tightly connected to the autonomic nervous system via viscerofugal neurons that project out of the gut to connect to the sympathetic nervous system with direct feedback to the ENS to modulate motor activity (18). Separate from sympathetic pathways, the ENS communicates with the central nervous system (CNS) via the vagus and pelvic nerves (19). These nerve fibers are heterogeneous (20, 21), receive input from distinct sensory cells (22) and other neurons, and interact with ascending pathways. Consequently, gut-to-brain and brain-to-gut pathways involve interconnected networks of different sensory cells, neurons, interneurons, and glia.

### Hormonal regulation of the gut-brain axis.

**Figure 1:**
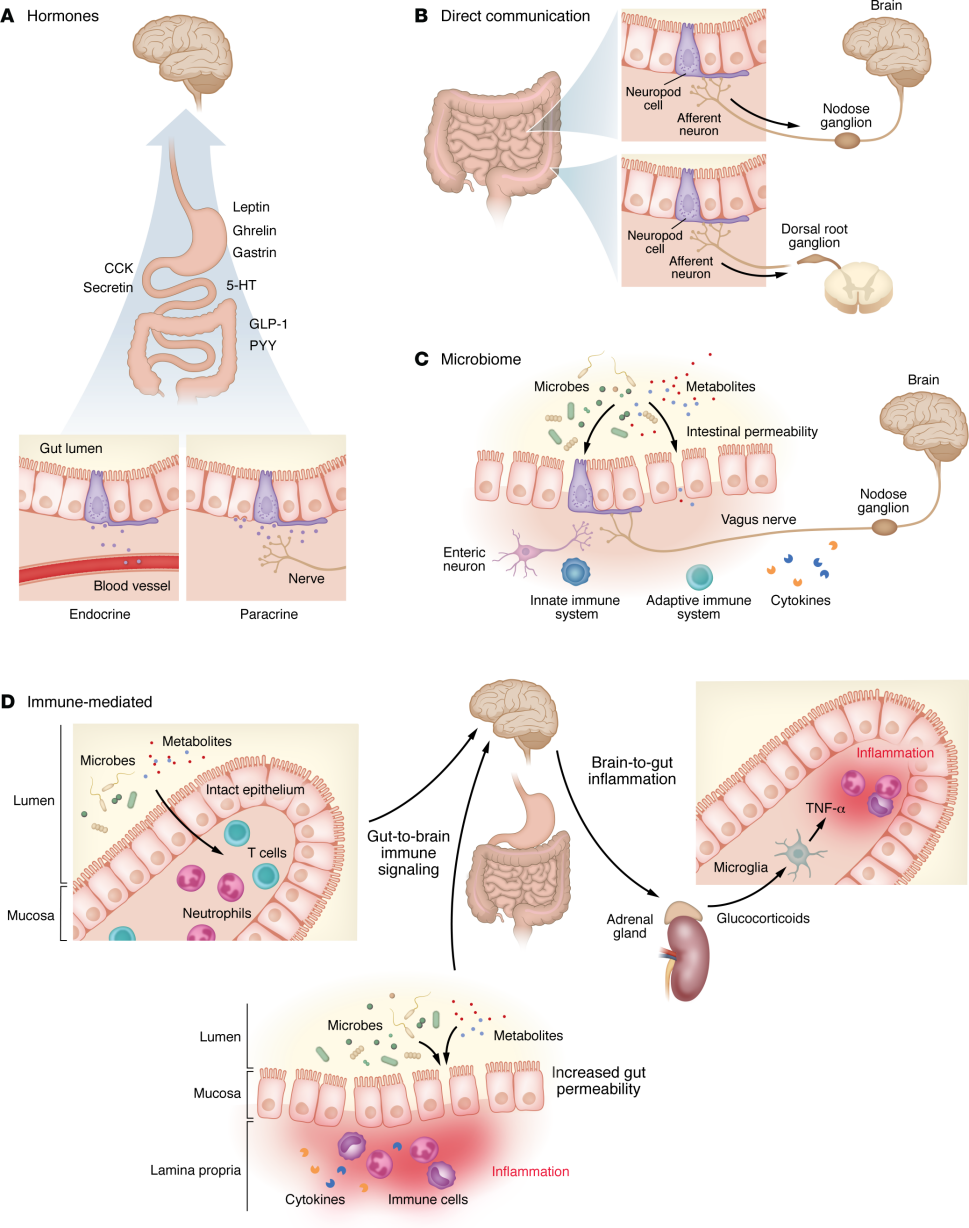
Mechanisms of signaling between the gut and the brain. Information can be transmitted from the gut lumen to the brain in a variety of ways, but recent research has highlighted four distinct categories of signaling. (**A**) In hormonal signaling, hormones from the gut epithelium are either released into the bloodstream (endocrine) or locally (paracrine), where they act via receptors to exert an effect. Hormones act on receptors in the ENS and CNS (particularly the hypothalamus) to receive these signals. 5-HT, 5-hydroxytryptamine (serotonin). (**B**) In neuropod-mediated signaling, EECs form close connections that rapidly transmit information from the gut lumen to the CNS. In more proximal regions of the gut (i.e., stomach, small intestine), signals are typically transmitted via the vagus nerve and convey nutritive information. Neuropod signaling in more distal regions (i.e., colon) conveys information related to visceral pain and stretch, which are received by the brain via the dorsal root ganglia. (**C**) Gut microbiota produce local effects in the gut lumen that affect epithelial permeability and allow transmission of the microbiota or associated metabolites into the bloodstream. Some of these changes induce an inflammatory response. Alternatively, microbes or metabolites (such as short-chain fatty acids) act locally on receptors to modify cell function. (**D**) The gastrointestinal immune system surveils the gut lumen with resident T cells and neutrophils that are activated by microbes and their metabolites and convey signals to the brain. Responses can be modified via inflammation within the gastrointestinal tract, leading to increased permeability and allowing further immune interactions. Concurrently, top-down mechanisms have been described, including glucocorticoid-dependent activation of ENS microglia leading to inflammation within the gut epithelium.

The gut is the largest endocrine organ and secretes more than 30 individual hormones (23). These hormones are central in coordinating digestion and are one of four known processes that facilitate communication between the gut and the brain (Figure 1A). Hormonal signaling from gut to brain originates in enteroendocrine cells (EECs) that make up a small fraction of the gut epithelium. EECs are functionally defined by their hormonal expression, which varies according to location in the intestine and distribution along the crypt-villus axis (24). While the current naming convention is largely alphabetical (i.e., L cells) and mostly unrelated to function, there have been calls to rename these cells according to hormone secreted and location (25). It is not uncommon for EECs to secrete multiple hormones (26), and recent studies using intersectional genetics highlight a heterogeneous assortment of EEC types with differential effects on feeding behavior and gut motility (27, 28).

While EECs can secrete hormones to affect digestive function in nearby organs (29), receptors for several gut hormones also exist in the CNS, including those for serotonin (30), cholecystokinin (CCK) (31), secretin (32), ghrelin (33), peptide YY (PYY) (34), and GLP-1 (35). Strikingly, some hormones have a more profound effect on gastrointestinal function when injected directly into the brain than when acting in the periphery (36). However, for peripheral hormones to exert a direct effect on the brain, they must overcome the blood-brain barrier. Hormones such as ghrelin and leptin are transported into the brain via specific transporter proteins (37, 38), but this is not a universal mechanism. In fact, serotonin, which exists as a neurotransmitter in the brain but is produced mostly in the gut (90% of total body content), does not cross into the CNS (39). In some cases, hormones can bypass the blood-brain barrier by binding to hormone receptors on circumventricular organs such the area postrema and subfornical organ (40). Alternatively, peripheral hormones can modify CNS activity through release of cytokines and nitric oxide from the blood-brain barrier itself (41).

Many of these same hormones can also act through peripheral nerves that signal to the brain. Notably, signal transduction through nerves such as the vagus, as well as afferent fibers with cell bodies in the dorsal root ganglia, represent a fundamental mechanism by which the CNS can receive information from the gut. In mice, the vagus nerve innervates the length of the gastrointestinal tract and contains receptors for hormones, including GLP-1, PYY, serotonin, and CCK (42). Vagal innervation is the greatest in the proximal intestine and declines across the length of the gastrointestinal tract, with possible differences in extent of innervation between human and mouse (43).

### Direct connections.

In the past decade, evidence has emerged that the gut communicates with peripheral nerves in a way that is distinct from hormonal signaling (Figure 1B). While it has been known for some time that EECs are electrically excitable (44), the discovery of neuropod cells, specialized EECs that contain podocyte-like processes near afferent nerve terminals and contain all necessary machinery for neurotransmission (45), suggested that this electrical excitability could convey information via a synaptic mechanism. Enterochromaffin cells are a large subset of EECs that are also electrically excitable, in close contact with nerve fibers, and can utilize neurotransmitters such as serotonin to directly modify signaling to communicate with the brain (46). Retrograde tracing techniques showed connections between neuropod cells and the brain in as little as a single synapse (47). However, recently it has been suggested that the distance between EECs and vagal and spinal afferents is too large to be classified as a synapse (48, 49), or that only a small subset of EECs form synaptic connections (50). Regardless, whether synaptic or paracrine, an EEC-neuronal connection distinct from hormonal signaling is a key mechanism of gut-brain communication.

### The microbiome.

Within the gut, the microbiome can influence brain function (Figure 1C). Microbiome composition is linked to diet (51) and can affect health (52–54). Gut microbes influence the CNS through hormone release, cytokine signaling, neurotransmitters, and release of bacterial byproducts that can either act within the gut or enter the systemic circulation (55). The microbiota and their metabolites have been shown to directly affect EECs (46, 56, 57), EEC-dependent activation of the vagus nerve (58) or sympathetic pathways (46), and hormone release (59). Thereby, the microbiome can modify gut-to-brain pathways to affect digestive function, CNS activity, and states of disease.

### The immune system.

Inflammation in peripheral organs, including the gastrointestinal tract, is linked to proinflammatory changes in the CNS (60, 61). Even in health, the gastrointestinal tract contains abundant immune cells that surveil the intraluminal contents (Figure 1D) (62, 63). In disease, cytokines can alter the permeability of the gastrointestinal epithelium, leading to exposure of gastrointestinal immune cells to alimentary contents (64). This is colloquially referred to as a “leaky gut,” and while the clinical implications of this are likely grossly overestimated (65), inflammation in the gastrointestinal tract can affect immune pathways and in turn gut-brain immune mechanisms. For example, a population of gut-derived T cells that are transcriptionally and functionally distinct from meningeal T cells migrate from the gastrointestinal tract to the paraventricular subfornical organ to regulate CNS homeostasis (66). Peripheral inflammation within the gastrointestinal tract can also be encoded by the brain. One study showed re-activation of gut-inflammation-responsive neurons in the insula reproduced gastrointestinal inflammatory patterns similar to the original peripheral insult (67). Taken together, these studies illustrate a role for immune cells in communicating signals from the gut to the brain and modifying CNS activity to respond to gut contents.

## Gut-brain mechanisms of digestive disease

### Disorders of gut-brain interaction.

Over 40% of people worldwide are estimated to have a disorder of gut-brain interaction (DGBI) (68), which includes IBS and functional dyspepsia and negatively impacts quality of life (69). Previously referred to as functional gastrointestinal disorders, DGBIs were renamed by the Rome IV criteria in May 2016 to recognize underlying gut-brain pathophysiology (70). These conditions are unique in gastroenterology in that serology, imaging, and endoscopy in DGBIs are normal without any characteristic microscopic features on biopsy to establish a clinical diagnosis. Therefore, diagnosis of DGBI is based on symptom pattern with exclusion of alternative processes.

Clinically, the manifestations of DGBI are varied and include multiple different organs (esophagus, stomach, colon, biliary system), with symptoms ranging from dyspepsia to constipation and diarrhea. Multiple studies have shown frequent overlap between DGBI subtypes (i.e., IBS and functional dyspepsia) in the same patients and, when this occurs, the risk of comorbid psychiatric symptoms is greater (69, 71, 72). In fact, psychiatric comorbidities of DGBI are common (73), and psychiatric symptom scores are associated with a reduced likelihood to respond to any DGBI treatment, including neuromodulators (74).

Given this frequent overlap between DGBI and psychiatric symptoms, changes in circulating hormones, which affect both the gut and CNS, have been proposed to explain disease pathophysiology. For example, DGBIs are closely associated with psychological stress (75) to the extent that both adult stress and early life stress mouse models are used to simulate IBS (76). Accordingly, stress-dependent changes in corticotropin-releasing hormone (CRH) have been proposed as a mechanism for DGBIs by increasing intestinal permeability (77). Similarly, there is clinical and preclinical evidence that estrogen affects DGBI pathogenesis (78, 79), leading to the hypothesis that estrogen explains the higher prevalence of DGBIs in women (78, 80, 81).

In a study of postprandial hormone levels in patients with IBS and non-IBS controls, patients with IBS had increased postprandial gastrin and insulin and decreased postprandial ghrelin compared with non-IBS controls (82). This was associated with changes in gut motility, which is a common feature across numerous DGBIs. Other studies have identified changes in EEC abundance in IBS populations (83–85). However, the results of these studies are variable, and it is difficult to discern whether these effects are secondary to or causative of DGBI (86).

Pain is a central feature of many DGBIs and is felt to be secondary to underlying visceral hypersensitivity. In mice, chemogenetic activation and silencing of enterochromaffin cells with designer receptors exclusively activated by designer drugs (DREADDs) revealed that a serotonergic subset of EECs is directly involved in both the response to luminal irritants and the establishment of visceral hypersensitivity (87). Specifically, the effect of luminal irritants or colonic distension was mitigated when these cells were silenced, but activation of serotonergic enterochromaffin cells led to sex-dependent effects on visceral hypersensitivity via spinal afferents in the dorsal root ganglia (DRG). Interestingly, in this study, both increases and decreases in serotonin signaling promoted anxiety-like behavior. Other work has demonstrated similar phenomena, with a knockout of epithelial serotonin reuptake (increased serotonin) having opposite effects to epithelial blockade of serotonin synthesis (decreased serotonin), although these effects were dependent on the vagus nerve and not the DRG (88).

Enteroendocrine circuits in the small intestine have also been directly linked to visceral hypersensitivity in DGBIs, with selective knockout of the Gucy2c receptor on CCK-containing EECs increasing visceromotor response to rectal balloon distension (89). While CCK-containing EECs are present in more of the intestine in rodents than in humans, they are not present in the rectum (90) where the balloon was distended, suggesting the possibility of yet-to-be-identified circuits that facilitate small intestinal communication with other areas of the intestine. Interestingly, while knockout of the Gucy2c receptor in the intestinal epithelium affected p-ERK staining in the dorsal horn of the spinal cord (89), it is unknown whether the EECs containing the Gucy2c receptor also signal via the vagus to modify visceral pain.

Changes in the microbiome have been linked to disease subtypes across DGBIs (55, 91). Multiple studies demonstrate distinct microbial and microbe-associated metabolomic patterns in IBS (92–95). Interestingly, these features appear to correlate with known therapies. The microbial signature of IBS includes organisms that ferment carbohydrates (95); a diet low in fermentable sugars (low FODMAP diet) is a mainstay of IBS management, and adherence to a lowFODMAP diet appears to shift the microbial profile of patients with IBS toward that of non-IBS controls (94). Similarly, adherence to a combined Mediterranean and low FODMAP diet not only improved symptoms but reduced microbial byproducts: fecal short- and branched-chain fatty acids (96). Interestingly, related molecules (such as isovalerate; ref. 87) have been shown to affect visceral hypersensitivity through EECs. Together, these findings indicate that diet can promote specific microbial profiles that enhance/reduce production of microbial metabolites that affect gut-brain circuitry to elicit symptoms of IBS. Similar evidence for microbial changes exists in functional dyspepsia (97, 98), with a potential role for microbiome-induced changes in motility (98, 99) contributing to symptoms. Notably, microbial changes are closely associated with direct actions on EECs (99). Gut microbes may also promote EEC survival or proliferation, as fecal microbiota transplantation in IBS increases EEC abundance (85). However, to date, no unique microbial species has been proven causative in DGBI or been shown to modify individual EEC subtypes.

Although not fully established, multiple lines of evidence suggest a role for altered gut immunity in DGBIs. DGBIs are associated with increased intestinal permeability (77, 100) as well as low-level inflammation (101), with mast cells implicated as a possible driver of hypersensitivity in IBS (77, 102). Accordingly, stress-induced changes in food sensitivity, which overlap with the clinical features of IBS in many patients, induce pain in a mast cell–dependent matter (103). In experimental models, colonic inflammation stimulated monocyte and neutrophil migration to the brain, leading to anxiety-like behavior (104), a common clinical finding in IBS. Together, these studies indicate that DGBIs may involve changes in gut permeability due to low-level inflammation that either directly influences CNS activity or modifies gut-to-brain pathways.

### Inflammatory bowel disease.

Inflammatory bowel disease (IBD) is characterized by two disease phenotypes, Crohn’s disease and ulcerative colitis, that produce chronic inflammation in the intestine. As an autoimmune disease, the pathophysiology of IBD is not directly linked to changes in gut-brain signaling. However, recent studies have illustrated that gut-brain pathways play an important role in gut inflammation.

There is growing evidence that psychological stress promotes inflammation in the periphery (105). In turn, peripheral inflammation, as seen in IBD, can expose the CNS to inflammatory signals via stress-related breakdown of the blood-brain barrier (106). Clinically, perceived stress in IBD is associated with worse disease outcomes (107). To evaluate the role of stress-induced exacerbations in IBD, a recent study combined psychological stress with dextran sodium sulfate–induced colitis in mice (108). As in humans, psychological stress worsened colitis severity. This effect was mediated by CRH-dependent glucocorticoid release that prompted glial cells within the ENS to stimulate local monocyte populations to produce TNF-α. TNF-α is closely linked to IBD, and TNF-α inhibitors such as infliximab are a first-line biologic therapy for the disease. Chronic stress may also reduce the ability of the intestinal epithelium to regenerate, as chronic stress affects signaling from the dorsal motor nucleus of the vagus to the ENS, reducing stemness in intestinal crypts (109). Consequently, while IBD is not caused by abnormal gut-brain signaling, increased brain-to-gut activation of stress pathways exacerbates inflammation and reduces epithelial cell regeneration, leading to worsened clinical outcomes.

In addition to brain-to-gut pathways, EECs are important contributors to barrier function and gut immunity (110, 111). Therefore, changes in these cell populations could affect gut inflammation in IBD. Studies in both IBD patients (112) and mouse models of colitis (113) have identified increased EEC abundance with distinct hormonal changes in states of disease (114). For example, ghrelin levels are higher in active IBD than either quiescent IBD or healthy controls and have been proposed as part of a biomarker equation to noninvasively evaluate disease flares (115). GLP-1, which is increased in active IBD, normalizes upon disease quiescence (116). Increased vagal signaling is associated with an antiinflammatory response in the gut (117), and vagal tone is decreased in IBD (118). However, the specific efferent pathways driving this reduction in inflammation have yet to be identified, and while vagal stimulation has been proposed as an IBD therapy (119), clinical trials have not moved beyond the proof-of-concept stage.

Proinflammatory microbial changes are well described in IBD (120–122). The microbiome is closely linked to pro- and antiinflammatory states, which may be dependent on long-term dietary habits (123). Consequently, current guidance from the American Gastroenterological Association suggests that patients with IBD follow the Mediterranean diet (124), which is associated with microbial changes that reduce inflammation (125). Specific microbial changes in IBD have not been well established due to heterogeneity among studies (126). Even in studies of the same patient population, disease flares are associated with temporal variability in the microbiome signature (127), complicating global conclusions. Therefore, there is a need to better define gut microbiome changes and their upstream signaling changes, including the role of diet, to identify more specific therapeutic approaches.

In animal models of colitis, gut inflammation is present in specific neuronal populations in the insula, with re-activation of these neurons causing peripheral inflammation in the gastrointestinal tract (67). The exact mechanisms of this process are unclear, but studies suggest that colitis results in recruitment of immune cells to the brain (104) with the potential to directly affect brain activity (60).

### Gastrointestinal motility disorders.

Changes in the ENS or communication between the extrinsic nervous system and the ENS can lead to disorders across gastrointestinal organs, ranging from ineffective esophageal motility causing dysphagia, to dumping syndrome causing an autonomic response to carbohydrate-rich foods, to chronic constipation limiting regular defecation. Gastrointestinal hormones such as ghrelin and motilin are essential for gastric emptying (128, 129). In the small intestine, serotonin is critical to contraction and intermixing of digested contents (130), whereas GLP-1 can block normal peristalsis (131). In the colon, serotonin contributes to colonic motility (132). While direct hormonal actions on the gut could explain motility disorders independent of any communication with the brain, numerous neurological diseases, ranging from Parkinson’s disease (PD) to multiple sclerosis (MS), are associated with changes in gut motility (133). Accordingly, it is evident that CNS activity influences gut contractility and the ability to defecate. Experimentally in rats, stimulation of the locus coeruleus in the brainstem led to colonic contraction via noradrenergic and dopaminergic receptors in the lumbosacral defecation center (134), which is also affected by serotonin within the spinal cord (135). More recently, studies using DREADDs demonstrated top-down control of this defecation center from neurons in the brainstem that can modify both noxious stimuli and stress-induced defecation (136). Taken together, these studies demonstrate a role for the CNS in control of gut motility that may be involved in pathogenesis of motility disorders and represents a future target for therapeutics.

## A gut-brain mechanism for neurologic disease

### PD.

PD is a motor disorder characterized by rigidity, decreased movement, and imbalance. The pathophysiology of PD is complex but involves loss of dopaminergic neurons in the substantia nigra through an interplay of neuroinflammation and α-synuclein accumulation. In addition to motor deficits, gastrointestinal symptoms are common in PD (137). Patients with PD are at higher risk for dysphagia, gastroparesis, constipation, and intestinal pseudo-obstruction even when compared to other neurological diseases such as Alzheimer’s disease (138). While neurological dysfunction could disrupt known top-down modulators of intestinal contraction that are known to play a role in motility (134), there is increasing evidence that the gut and associated gut-brain signaling are directly involved in the pathogenesis of PD. In support of this concept, epidemiological studies revealed that complete truncal vagotomy, but not the more limited highly selective vagotomy, was associated with reduced risk of PD (139).

α-Synuclein is produced in both the CNS and peripheral nervous system, including the gut, and increases in response to enteric infection (140) or inflammatory colitis (141). The Braak hypothesis postulates that PD pathology spreads in a predictable and progressive pattern (142), leading to the concept that α-synuclein spreads in a prion-like fashion from the gut to the brain via the vagus nerve (143, 144). In mice, injection of α-synuclein fibrils into the duodenal and pyloric muscularis induced α-synuclein pathology in the dorsal motor nucleus of the vagus and later other regions of the brain, including the substantial nigra (145). Vagotomy prevented this gut-to-brain spread. EECs contain α-synuclein (146), and by virtue of their connection with the vagus nerve, this established gut-to-brain circuit represents a possible pathway for the origin of PD. However alternative mechanisms of gut-to-brain transfer, including via immune cells, have also been proposed (147). A link between gut pathology and PD development in humans has also been shown, as patients with upper endoscopic findings of mucosal tears are more likely to develop PD later in life (148). However, it remains unclear whether this is causative or correlative, as it is unknown whether these tears lead to accumulation of α-synuclein or allow existing α-synuclein to transfer to the brain. Of note, the gut-to-brain model of PD is not without controversy, as there are also data to support brain-to-gut spread (120).

Within the gut, deposits of α-synuclein can have local consequences. In mice, transfer of human HLA-DRB1*15:01 and subsequent exposure to α-synuclein–derived epitopes leads to intestinal inflammation, a loss of enteric neurons, constipation, and weight loss (149). These data, combined with evidence of gastrointestinal inflammation leading to PD development (140, 141), support a possible neuroimmune role of PD pathogenesis. Thus, the clinical manifestations of PD in the gut, which can occur years before onset of motor symptoms, could be due to an inflammatory response to accumulating α-synuclein prior to CNS spread through the vagus. The microbiome may also play a role in this process, as the microbial profile of PD patients is distinct (150) and involves a preponderance of proinflammatory organisms, including those that dysregulate neuronal signaling and promote α-synuclein production (151).

### MS.

Although not as robust as PD, there is increasing evidence that changes in gut-brain signaling might also play a role in MS. MS is characterized by neuronal demyelination, leading to progressive neurologic dysfunction. MS is also associated with constipation. In experimental models of MS, autoantibodies against ENS and glia slowed intestinal transit (152).

The microbiome may also play a role in MS pathogenesis. In a study of 576 MS patients and 1152 household controls, patients with MS were found to have a distinct microbial and microbial metabolite profile that varied in response to disease-modifying treatments (153). Some studies have argued that the altered microbiome in MS causes disease (154), and others attempting to modify the microbiome have shown some success on improved disease course (155). The gut may also play a direct role in MS pathogenesis by initiating inflammatory pathways. Viewed by intravital imaging, autoreactive encephalitogenic T cell populations are activated in the lamina propria of the small intestine and subsequently adopt a Th17-like proinflammatory phenotype (156). This profile appeared to be dependent on both the microbiome and MHC II, as germ-free mice or MHC II antibodies greatly mitigated the inflammation. In support of a role for gut inflammation in initiating disease, molecular MRI evaluation of intestinal inflammation correlated with disease severity in a mouse model of autoimmune encephalitis (157). Together, these data suggest that MS may be dependent on gut inflammatory pathways, potentially in concert with the microbiome.

## A gut-brain mechanism for psychiatric disease

### Depression.

The World Heath Organization estimates that 5.7% of adults worldwide suffer from depression (158). Depression, as well as depression-like symptoms, are closely comorbid with DGBIs (73), and changes in gut signaling pathways can produce anxiety and depression-like behavior (87, 88). Depression has also been linked to the microbiome, and fecal microbiota transplant from patients with depression to germ-free mice was sufficient to induce a depression-like phenotype (159) as well as an inflammatory hippocampal gene expression pattern (160). While CRH release following stress has been related to DGBI symptoms through increased intestinal permeability (77), this same permeability has been linked to social avoidance in a chronic social defeat stress model of depression (161). Gut-brain therapies have been attempted for major depressive disorder (MDD), including FDA-approved vagal stimulation for resistant disease (162), with recent clinical trials demonstrating at least some benefit (162). Thus, MDD pathophysiology may include top-down stress-responsive changes in intestinal permeability that facilitate intraluminal metabolites and the microbiome to affect CNS function, potentially involving the vagus nerve.

### Schizophrenia.

Like MDD, schizophrenia is associated with an altered gut microbiome profile and proinflammatory signaling pathways. Genome-wide association studies have demonstrated that schizophrenia and gastrointestinal diseases are closely related and share common gene variants linked to immune system function (163). Similarly, gut microbiome changes in patients with schizophrenia are closely related to proinflammatory pathways (164), although some multiomics analyses find less of an effect of the microbiome and more amino acid and lipid metabolism pathways affected (165). The mechanisms explaining these contributions are currently unknown.

## Gut-brain mechanisms for obesity

Gut-brain signaling is essential to ingestive behavior. In 1950, a spontaneous mutation at The Jackson Laboratory led to the production of *ob/ob* mice, which exhibited weight up to four times that of a standard mouse (166). However, it was not until four decades later that the responsible gene mutation and its hormonal product, leptin, were identified (167). The discovery of ghrelin in rat stomach (168) led to the idea that opposing hormones modulate energy balance, with leptin promoting satiety and ghrelin stimulating food intake. A logical extension of this reasoning was that obesity resulted from excess ghrelin and reduced leptin. However, the relationship with BMI in patients is actually the opposite (positive correlation of BMI with leptin and negative correlation of BMI with ghrelin) (169). Furthermore, ghrelin, not leptin, increases with weight loss (170). This understanding led to the concept that resistance to these hormones exists in states of obesity (171). Another gut hormone, GLP-1, is secreted at lower levels in obese individuals (172, 173) and there is some evidence from human data favoring increased postprandial GLP-1 following weight loss (174). In addition, postprandial GLP-1 levels might play an important role in the success of bariatric surgery (175). Thus, hormonal signaling pathways appear to be related to obesity, but the exact contributions of each hormone are still being determined.

Recently, there has been increased recognition of the role of a direct gut-brain connection in guiding food intake. Diversity of both EECs as well as the vagal sensory neurons has been linked to specific components of feeding behavior (20, 28). Functionally, cells of the right vagus nerve form circuits with dopaminergic neurons in the substantia nigra that, when stimulated, facilitate a conditioned place preference toward the stimulated side (176). Consequently, activation of vagal afferents is rewarding, likely through dopaminergic release in the brain. This aspect of reward is essential to our understanding of obesity, as the high caloric macronutrients sugar (47, 177) and fat (178) have been shown to activate vagal pathways. While the vagal neurons involved in fat and sugar sensation are distinct, the combined ingestion of fat and sugar leads to more dopamine release in the nigrostriatum than either fat or sugar alone (179). Thus, the Western diet, which is high in both fats and sugars, might promote overeating and obesity through an additive effect on gut-brain reward pathways. In addition, neuropod cells activate the vagus nerve in response to intraluminal sugars via the neurotransmitter glutamate (47). These same cells respond to artificial sweeteners via a different neurotransmitter (ATP), and guide preferences for sugar over artificial sweetener following preconditioning (180). These findings suggest that direct gut-brain connections play a role in food choice.

Unsurprisingly, changes in the microbiome have been associated with obesity (181, 182). This is likely due at least in part to underlying differences in diet, which help to shape the microbiome (51). However, there are likely both causal and reactive components of the gut microbiome, so understanding the microbiome may assist in developing personalized interventions for weight loss (183). Accordingly, there is evidence for the microbiome-modulating efficacy of specific dietary strategies (184). Albeit with the same limitations of causation due to dietary changes, changes in the microbiome have been described in other eating disorders such as anorexia nervosa, bulimia nervosa, and binge eating disorder (185), with some evidence suggesting that altered microbial proteins can induce autoantibodies that affect neuroendocrine signaling (186).

## Pharmacological targeting of gut-brain pathways

**Figure 2:**
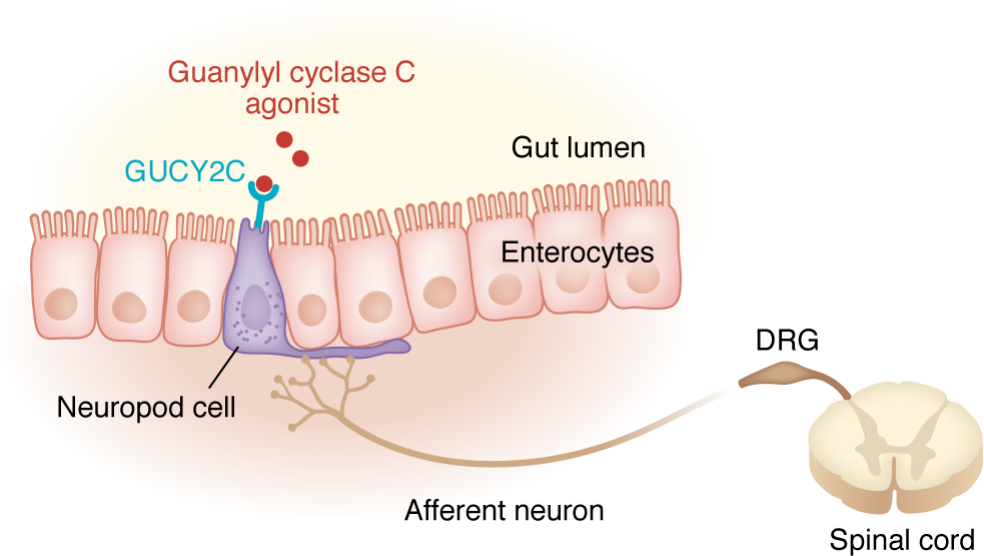
Example of gut-brain directed pharmacotherapy. Guanylyl cyclase C agonists (e.g., linaclotide) bind to the GUCY2C receptor on neuropod cells to inhibit sensory neurons of the dorsal root ganglion and reduce visceral pain in IBS (89).

The extent of current pharmacologic agents that act on gut-brain pathways is unknown. However, two widely prescribed medications have been shown to be at least partially dependent on gut-brain mechanisms. Guanylyl cyclase C agonists have emerged as a target for visceral pain, with the finding that FDA-approved agonists such as linaclotide reduce pain in IBS. In an analysis of four separate randomized control trials, over 50% of patients with IBS with constipation treated with linaclotide reported at least a 30% reduction in abdominal pain (187). An explanation may lie in the discovery that GUCY2C neuropod hyperexcitability was reduced by linaclotide in vitro and correlated with reduction in visceral pain in vivo (Figure 2) (89). These data strongly suggest that directly targeting gut-brain signaling pathways via neuropod cells could help mitigate symptoms of visceral pain in DGBIs.

Since receiving FDA approval for treatment of obesity in 2014, GLP-1 receptor agonists have become a mainstay of obesity therapy. Recent evidence suggests these medications also may be effective in other brain-based disorders, including Alzheimer (188) and alcohol use disorder (189). In obesity, these drugs are highly effective, with some formulations achieving mean weight loss of over 20% in 72 months of follow-up (190). As GLP-1 is a gut hormone with receptors on the vagus (42), in the brain (35), and circumventricular organs (40), these medications directly mimic endogenous gut-brain incretin hormone pathways. GLP-1 has long been known to promote satiety and reduce food intake (191), although there is increasing evidence for accelerated metabolic activity (192). The exact mechanisms driving efficacy of GLP-1 receptor agonists in obesity are unknown, as the effects are widespread and include delayed gastric emptying and changes in blood glucose (193). Endogenous GLP-1 release within the brainstem reduces eating (194). While the vagus nerve contains neurons with receptors for GLP-1 as well as neurons that act on the brainstem to facilitate endogenous GLP-1 release, these two processes do not appear to overlap. Indeed, the vagal neurons that facilitate endogenous release of GLP-1 in the brainstem contain receptors for oxytocin but not GLP-1 (195). This suggests that there are parallel peripheral and central GLP-1 pathways that independently suppress appetite that could be simultaneously coopted for additive weight loss effects in humans. Accordingly, small studies using medications to target the brainstem preproglucagon neuronal pathway in concert with GLP-1 receptor agonists that do not target this pathway show promising effects (196).

## Conclusion

Connections between the gastrointestinal tract and nervous system relay information about food, metabolites, and irritants within the gut lumen. While essential for homeostatic function, these pathways are altered in disease, with varying clinical presentations ranging from abdominal pain in IBS, to psychological distress in MDD, to constipation in PD. New technologies have enabled better understanding of gut-brain interactions, as well as how these pathways are implicated in disease and disease-modifying therapies. However, critical challenges lie ahead, including facilitating reproducibility, especially as it pertains to microbiome studies, translating studies to humans, and better clarifying the differing roles of rapid neuropod-based, paracrine, and hormonal EEC-based activities.

Nonetheless, our understanding of gut-to-brain signaling and its role in inflammation, stress, and metabolism has greatly expanded over the past decade. It is expected that future advances will lead to additional understanding of disease states and novel gut-brain–targeted therapeutics.

## Funding support

This work is the result of NIH funding, in whole or in part, and is subject to the NIH Public Access Policy. Through acceptance of this federal funding, the NIH has been given a right to make the work publicly available in PubMed Central.

- NIH grants F32 DK142208 and T32 DK007568 (to ZSL).
- NIH grants DK124474 and DK120555 (to RAL).
- Aligning Science Across Parkinson’s award ASAP-020527 through the Michael J. Fox Foundation for Parkinson’s Research (to RAL).

## Figures and Tables

**Table 1:**
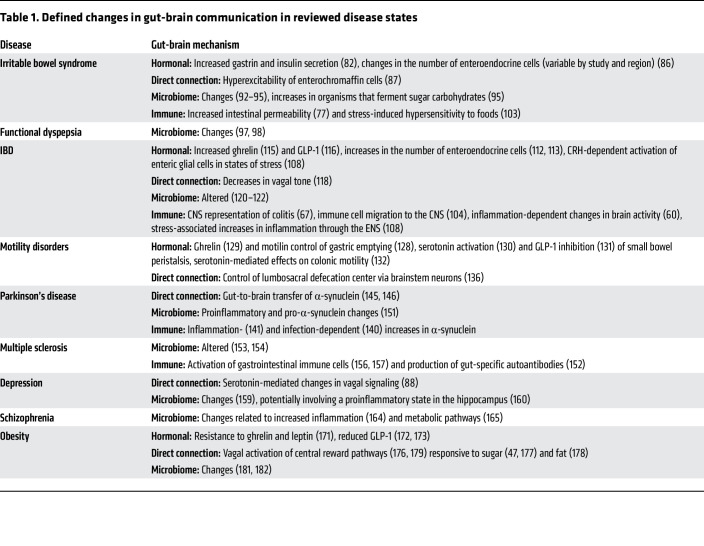
Defined changes in gut-brain communication in reviewed disease states

## Version 1 01/02/2026

Electronic publication
